# Temperature-dependent photoluminescence properties of porous fluorescent SiC

**DOI:** 10.1038/s41598-019-52871-6

**Published:** 2019-11-08

**Authors:** Weifang Lu, Abebe T. Tarekegne, Yiyu Ou, Satoshi Kamiyama, Haiyan Ou

**Affiliations:** 1 DTU Fotonik, Technical University of Denmark, Ørsteds Plads, Building 345, DK-2800 Kgs., Lyngby, Denmark; 2 Department of Materials Science and Engineering, Meijo University, 1-501 Shiogamaguchi, Tenpaku-ku, Nagoya 468-8502 Japan

**Keywords:** Optics and photonics, Optical materials and structures

## Abstract

A comprehensive study of surface passivation effect on porous fluorescent silicon carbide (SiC) was carried out to elucidate the luminescence properties by temperature dependent photoluminescence (PL) measurement. The porous structures were prepared using an anodic oxidation etching method and passivated by atomic layer deposited (ALD) Al_2_O_3_ films. An impressive enhancement of PL intensity was observed in porous SiC with ALD Al_2_O_3_, especially at low temperatures. At temperatures below 150 K, two prominent PL emission peaks located at 517 nm and 650 nm were observed. The broad emission peak at 517 nm was attributed to originate from the surface states in the porous structures, which was supported by X-ray photoelectron spectra characterization. The emission peak at 650 nm is due to donor-acceptor-pairs (DAP) recombination via nitrogen donors and boron-related double D-centers in fluorescent SiC substrates. The results of the present work suggest that the ALD Al_2_O_3_ films can effectively suppress the non-radiative recombination for the porous structures on fluorescent SiC. In addition, we provide the evidence based on the low-temperature time-resolved PL that the mechanism behind the PL emission in porous structures is mainly related to the transitions via surface states.

## Introduction

Silicon carbide (SiC) has excellent electrical, mechanical and thermal properties because of its strong covalent bonds resulting from its inherent wide indirect bandgap and valence-band edge at low energy^1,2^. These unique properties enable SiC and its related nanostructures to be an ideal material for high power electronics^3^. As a material that is non-toxic with a high elastic modulus, low friction coefficient and high resistance to corrosion in harsh environments, SiC is also an ideal material for biomedical applications^4–8^. In addition, its UV light absorption and transparency for visible light allow it to be an ideal material for optically based biosensors or photodetectors at UV wavelengths^9,10^. Porous SiC has been proven to be favorable as a highly promising sensing material due to its large internal surface area that facilitates high activity in surface reactions^11,12^. A significant advantage of porous SiC is that the porous structure can accommodate strain and threading dislocations to yield high quality epilayers^13–15^. Furthermore, porous SiC can yield remarkable blue-green photoluminescence (PL), which may expand their application to the field of lighting^16–21^. It has been demonstrated that nitrogen and boron co-doped 6H-SiC (fluorescent SiC) exhibits high-efficiency yellowish light emission^22^. Meanwhile, efforts has been made towards realizing white light emission by using a simplified hybrid fluorescent SiC structure with a porous surface layer^23^. A color rendering index as high as 81 has been achieved, and our previous results demonstrated the feasibility of tuning the emission peak and intensity of porous fluorescent 6H-SiC by controlling the thickness of the porous region and certain parameters of the surface passivation process^24^. It has been demonstrated that the emission in SiC quantum dots and nanoparticles is related to surface defects^25,26^, which has much smaller size than porous structures. Likewise, one possible explanation of the photoluminescence properties of porous SiC is related to the diversity in surface states^21^; therefore, it is important to know the chemical groups of the internal surface on the porous SiC. It has been demonstrated that the surface of porous SiC contains a wide variety of different chemical groups: carboxylic acid groups, silane groups, hydrocarbon fragments and hydroxyl groups^20,27,28^. Since it is important to understand the underlying physics of luminescence phenomena for its engineering applications, it is critical to carry out a thorough analysis of the carrier recombination mechanisms in porous SiC structures. Temperature-dependent PL is one of the standard methods to characterize the emission properties of semiconductor materials. It can provide information about the defect-related carrier transport dynamics^29–31^. However, to date, there are few works reported on the temperature-dependent optical properties of porous SiC with or without B-N dopants^32^. In addition, the effect of passivation on PL properties of porous SiC has never been investigated at low temperatures.

In this work, we report the passivation effect on porous fluorescent SiC by analyzing temperature-dependent and time-resolved PL spectra acquired in a temperature range between 64 K and 290 K. The PL quenching mechanisms were explored by comparing the temperature-dependent integrated PL intensity and PL decay curves of the porous SiC. Through this work significant insight was built from these investigations on the PL phenomena of porous SiC with surface passivated by atomic layer deposited (ALD) Al_2_O_3_ films. We provide evidence based on the low-temperature time-resolved PL that the mechanism behind the PL emission in porous structures is mainly related to the transitions via surface states.

## Experiments and Results

### Porous SiC fabrication and morphology characterization

The fluorescent SiC samples were lab-grown using a fast sublimation growth process (FSGP) at 1900 °C with N and B doping concentration of 1.3 × 10^18^ cm^−3^ and 0.9 × 10^18^ cm^−3^, respectively. A commercial 6H-SiC substrate (with background doping of N) from SiCrystal AG (Germany) was used for comparison. Firstly, a 100 nm thick nickel layer followed by a 10 nm thick titanium layer and a 200 nm thick gold film was deposited on the back side of the SiC samples as a back contact. Porous SiC samples with a typical pore diameter around 30–50 nm were prepared using an anodic oxidation method. The anodic oxidation was processed under pulsed-current to reduce the adhesion of CO/CO_2_ reaction product bubbles to the surface. Detailed processing and typical scanning electron morphology of porous structures can be found in our previous work^23,24^. Two porous samples (commercial 6H-SiC: sample a and fluorescent 6H-SiC: sample b) were cut into two pieces (a_1_, a_2_, b_1_, b_2_), respectively. The thicknesses of the porous layer in samples a and b are 13 µm and 10 µm, respectively. Samples a_2_ and b_2_ were passivated with Al_2_O_3_ deposited by ALD (Model R200, Picosun, Finland) in order to suppress the non-radiative recombination. According to the optimized passivation condition for porous SiC^24^, 200 deposition cycles were conducted to form a 20 nm Al_2_O_3_ layer on the surface of the porous structures. Post-deposition annealing was performed at 350 °C for 5 min in a N_2_ ambient.

Transmission electron microscopy (TEM) was used to investigate the structural and chemical distribution of sample b_2_. The specimens were prepared perpendicular to the bulk using an *in-situ* lift-out technique by a dual-beam focussed ion beam (FIB)/SEM (FEI Helios EBS 3) equipped with a gallium (Ga) ion source and a micromanipulator. The lift-out technique in this equipment allows for rapid TEM specimen preparation with minimal mechanical damage. A protective layer of platinum (Pt) was deposited onto the preselected area of the porous layer using FIB in order to protect the surface of porous layer from the later FIB-induced damage during the milling process. An electron-transparent thin lamella (10 × 5 × 0.150 µm) can be milled using focused Ga ion sputtering operating at an accelerating voltage of 30 kV. At the final step of the preparation, the specimen surfaces (including Pt) were cleaned by a 2 kV Ga ion beam to minimize the damage caused by the FIB-thinning step. The ultra-thin lamella was removed and placed on a copper TEM grid using the micromanipulator. The FIB lamella preparation only consumes a small volume of porous SiC, leaving most of the sample unaffected by the sputtering. TEM images and elemental maps were acquired at 300 kV in a field-emission-gun TEM system (FEI Titan 80-300ST TEM), equipped with a solid state EDX detector.

**Figure 1:**
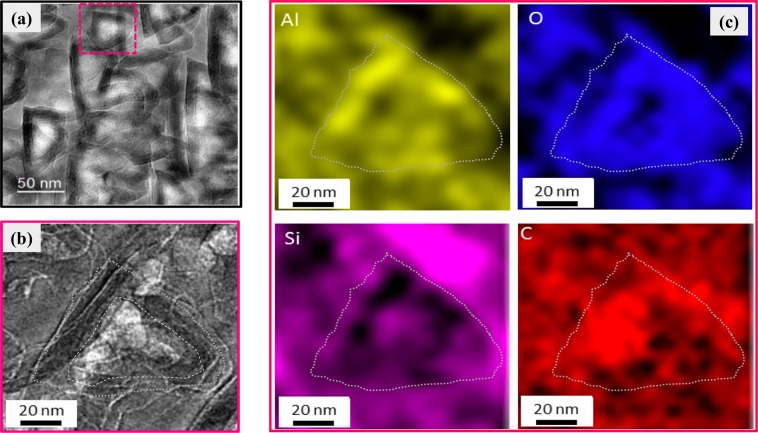
(**a**) Cross-sectional TEM images of porous sample b_2_ near the middle layer with surface passivation. (**b**) An enlarged image around a pore, marked by dotted white line. (**c**) The TEM-EDX elemental maps (Al, O, Si, C) around the pore corresponding to (**b**).

The authors Shishkin *et al*. have clarified that, during anodic oxidation, the charge near the surface of the SiC substrate redistributes, resulting in three distinguished regions of potential drops^33^. The space charge region in the SiC bulk due to ionized donors, is inversely proportional to the doping concentration, and the theoretically calculated width of the space charge layer is 30–50 nm. Figure 1(a) shows the TEM image acquired from the middle area of the layer, while several pores pile up perpendicularly to the image. The triangular pores with a dimensional size around 50 nm can be clearly seen, as shown in Fig. 1(b). The morphology of porous SiC is very sensitive to the exposed crystalline face to the etching, particularly, the etching rate on the C-face is faster compared to the Si-face^33^. Generally, the oxidation is much slower on Si-rich surfaces of SiC than the C-rich surface^33,34^. Consequently, the surfaces which make up the sidewalls of the triangular pores are Si-rich etch-stop surfaces.

On the other hand, the boundaries between the SiC and Al_2_O_3_ film is quite obvious. The area around one pore was characterized by EDX mapping, as shown in Fig. 1(c), where elemental mapping revealed Al (yellow), O (blue), Si (purple), and C (red) distributions. From the element mapping results, it is seen that the pore area (marked by dashed grey lines) comprises of not only Al and O but also a large amount of C, while Si is mainly distributed outside of the pore area. The C signal in the pore area is attributed to the C-related defects, such as C–Si–O or Si–C–O, C–O and O–C=O bonds^35,36^. On the other hand, the detected depth of EDX in TEM system is 10–100 nm, so other adjacent pores or crystal part under the pore can also be detected, leading to a lack of distinct boundary of the elements distribution. Despite this, the EDX elemental maps can demonstrate the coverage of ALD Al_2_O_3_ film on the pores.

### X-ray photoelectron spectroscopy

**Figure 2:**
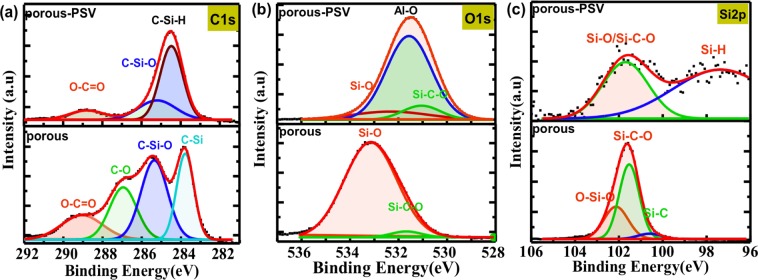
XPS core level spectra of (**a**) C1s, (**b**) O1s, and (**c**) Si2p in samples a_1_ (“porous”) and a_2_ (marked as “porous-PSV”).

X-ray photoelectron spectroscopy (XPS) was used to characterize the chemical composition of the porous SiC (samples a_1_ and a_2_). The porous samples a_1_ and a_2_ were measured using an Al K-Alpha radiation (Thermo Scientific) with pass energy of 50.0 eV. The reference C–C peak at energy of 284.4 eV was used for calibration. The measured high-resolution core level spectra of C1s, O1s and Si2p with Gaussian fitting are shown in Fig. 2. The C1s spectrum for porous SiC before passivation was fitted with four separate Gaussian components. The binding energies of the decomposed peaks are 283.7 eV, 285.3 eV, 286.7 eV and 288.7 eV, which are attributed to C–Si and surface reconstructions of C–Si–O or Si–C–O, C-O and O–C=O bonds^35,36^, respectively. After ALD passivation, the surface of the porous sample covered with a thin Al_2_O_3_ film. Thus, the composition ratios of C-related O bonds substantially decrease due to the limit probing depth of XPS, while the dangling bonds were saturated by H atoms and formed C–Si–H bonds^37^.

In Fig. 2(b), the deconvolution of O1s peaks confirms the existence of C–Si–O/Si–C–O bonds, which were generated during the anodic oxidation process. However, the penetration depth for XPS detection is only several nanometers, thus the O1s peak is dominated by the signal from Al-O bonds present in the passivating layer. The high resolution XPS spectrum of Si 2p in Fig. 2(c) shows a peak at 100.6 eV, demonstrating the existence of Si-C bonds in the porous structures. During the anodic etching process, the reaction product SiO_2_ was dissolved by HF solution. Afterwards, some of the exposed Si with dangling bonds were re-oxidized in air. In addition, peaks observed at 101.9 eV and 102.5 eV are indicative of Si-related oxidation products (C–Si–O/Si–C–O and O–Si–O, respectively). As mentioned in previous works, the Si dangling bonds can be passivated by H atoms in ALD deposited Al_2_O_3_ films. Thus, the appeared weak bonds in Fig. 2(c) might be related to Si-H bonds in porous SiC after ALD passivation^24^. It is known that the reaction between SiC and HF can produce a C-enriched phase at the surface and provide a high density of C-related surface defects (C-related O defects center: Si–O–C or C-related neutral oxygen vacancy: O_3_≡C–Si≡O_3_)^34,38,39^. Based on the binding energies reported by Shimoda *et.al*.^35^, the existence of silicon oxycarbide bonds and oxygen incorporations can be identified, i.e., the silicon atoms were bonded to carbon and oxygen atoms after electrochemical etching. All these C-related chemical groups are potentially the constituents of surface defects which would affect the optical properties of porous SiC. The results here are in agreement with the interpretation of the broad blue-green emission properties in porous SiC^23^, which is mainly attributed to non-bridging oxygen hole centers^40^ and C-related defect centers located near the interface^41,42^.

### Temperature dependent PL spectra

**Figure 3:**
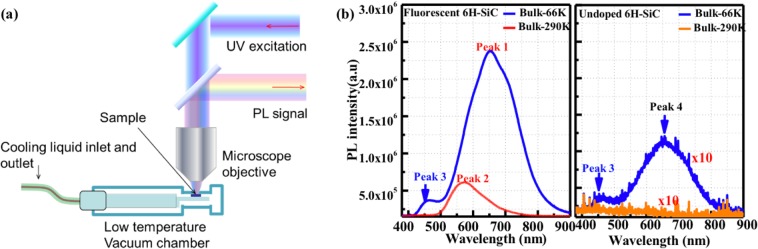
(**a**) Schematic setup for the temperature dependent micro**-**PL system. (**b**) PL Spectra of bulk fluorescent and commercial 6H-SiC measured at low (66 K) and room temperatures (290 K). The PL signal in commercial 6H-SiC was enlarged 10 times.

To assess the effects of the ALD passivation and surface defects upon the optical properties of porous SiC, temperature-dependent PL measurements were performed where the sample temperature was controlled using a cryostat system (micorstatHiRes, Oxford instruments). The sample temperature range was varied from 64 K to 300 K using liquid nitrogen as a coolant. The setup schematic of the micro-PL optical setup is shown in Fig. 3(a). The temperature was monitored by a MercuryiTC cryogenic environment controller (Oxford instruments), where the sensor is located close to the sample. A CW laser diode (center wavelength at 375 nm) was used to excite the sample at normal incidence. The size of the light spot focused on the samples was 0.148 mm^2^ with power of 24 μW. The PL signal was collected by a microscope objective and was fiber-coupled to an Andor spectrometer (SR-303I-A spectrograph with DU-420-0E CCD). To exclude the effect of the excitation peak on the PL, the PL signal was transmitted through a low pass filter with cut-off wavelength of 405 nm that was fitted in the microscope. The measured PL signals were acquired with an exposure time of 25 ms and the number of acquisitions was set to 300 to maintain a high signal-to-noise ratio.

Firstly, the PL characteristics of the bulk fluorescent and commercial SiC were investigated at room temperature and at 66 K, as shown in Fig. 3(b). From the low temperature PL spectra of fluorescent 6H-SiC substrate, a prominent peak at 650 nm (“Peak 1”) is observed, which is attributed to the N-B donor-acceptor-pairs (DAP) recombination. Another intense broad peak near 460 nm (“Peak 3”) might be ascribed to Al-N DAP with the unintentionally incorporation of Al atoms induced during the crystal growth^43,44^. The typical yellowish broad emission peak for fluorescent SiC can be observed near 572 nm (“Peak 2”) at 290 K. There is no obvious “peak 2” emission in the commercial 6H-SiC. However, the weak peak located at 656 nm (Peak 4) is quite similar to the “peak 1”, thus the commercial SiC may contain B contamination.

**Figure 4:**
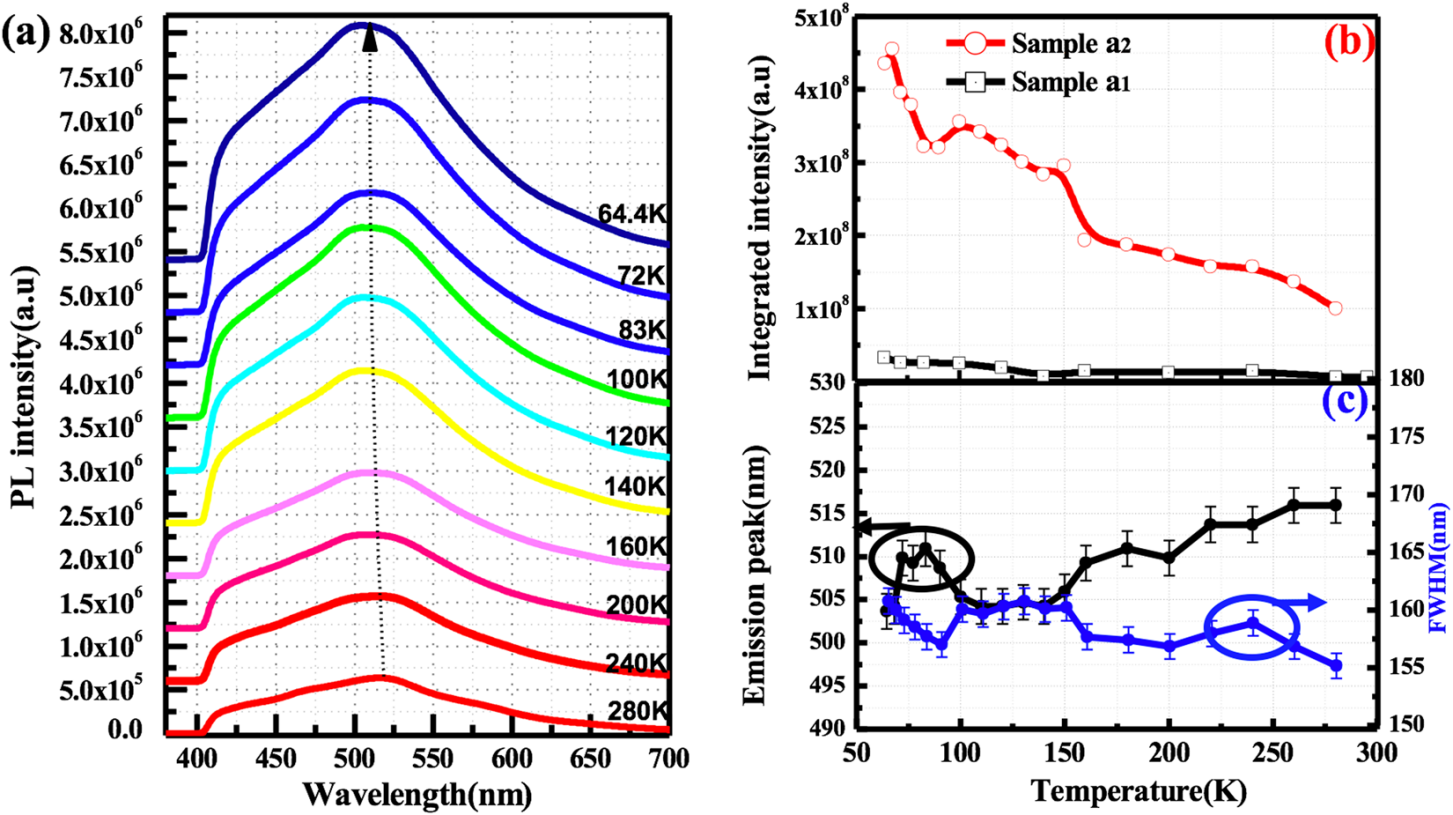
(**a**) The temperature-dependent PL spectra of sample a_2_ with surface passivated by an ALD deposited Al_2_O_3_ film recorded in the range from 64 K to 280 K. (**b**) The temperature-dependent integrated PL intensity of samples a_1_ and a_2_. (**c**) The emission peak and FWHM of sample a_2_ as a function of the temperature with an error bar of ±5%. The peak position and FWHM was nominally picked based on the maximum value of PL intensity.

In order to reveal the emission properties in porous structures, the temperature-dependent PL spectra of porous SiC with surface passivation was measured. Figure 4 shows the temperature-dependent PL spectra of porous sample a_1_ and a_2_ recorded in the range from 64 K to 280 K. The blue-green emission peak (~517 nm) here has been ascribed to the emission from non-bridging oxygen hole centers and C-related defect centers located near the interface^23^. It is clearly revealed in Fig. 4(b) that the integrated PL intensity of samples a_1_ and a_2_ decreases by 78% and 80%, respectively, when the temperature increases from 64 K to 280 K. The surface states are more likely to participate in the radiative recombination more efficiently at low temperatures, which can suppress the thermal activation of carriers^31^. In addition, the experimental results for porous commercial samples without surface passivation show the same emission peak. For comparison, the integrated PL intensity of sample a_1_ is plotted in Fig. 4(b). The remarkable enhancement of the luminescence intensity in the porous SiC can be explained by the efficient passivation of non-radiative surface states by ALD Al_2_O_3_ film as described in TEM-EDX and XPS characterization. According to Fig. 4(c), the peak wavelength slightly increased from 503 nm to 517 nm when the temperature increases from 64 K to 280 K, while the full width half maximum (FWHM) decreases. The slight red shifting of the emission peak can be ascribed to the change of contributions from the emission components of the surface states and the classical bandgap narrowing as with increasing temperature (32 meV)^45^.

**Figure 5:**
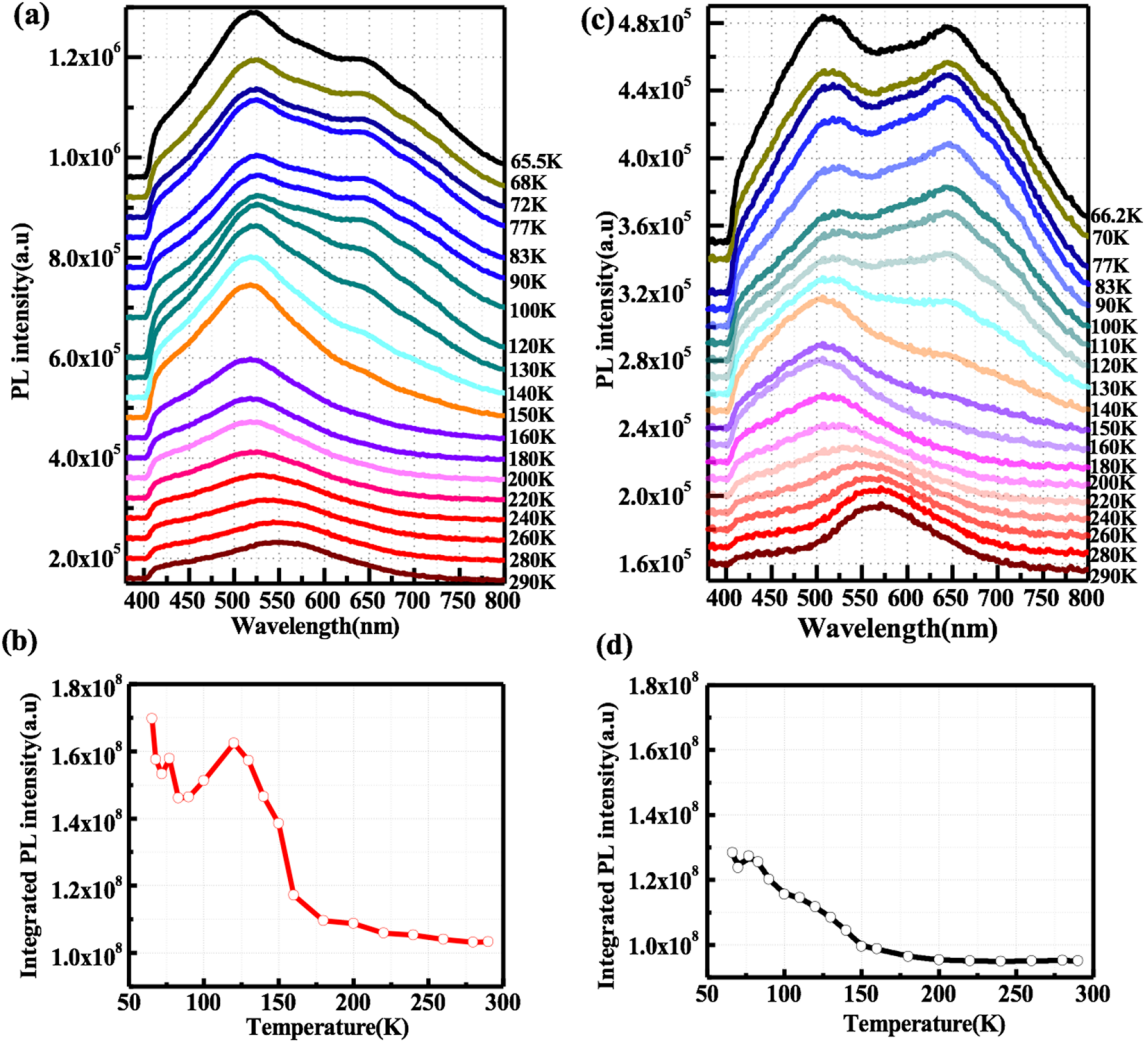
(**a**) The temperature-dependent PL spectra and (**b**) integrated PL intensity of the sample b_2_ with surface passivated by an ALD deposited Al_2_O_3_ film recorded in the range from 65 K to 290 K. (**c**) The temperature-dependent PL spectra of sample b_1_ without surface passivation. (**d**) The integrated PL intensity of porous SiC recorded as a function of the temperature in the range from 66 K to 290 K.

The temperature-dependent PL spectra of porous fluorescent samples with (b_2_) and without (b_1_) surface passivation are shown in Fig. 5. Compared with the temperature-dependent PL spectra of commercial sample a_2_ with surface passivation, it can be verified that the emission peak (~517 nm) is attributed to the surface defects from porous structures. A knee appears in the integrated PL intensity curves, *i.e*., the intensity gradually increases as the temperature increases, as shown in Fig. 5(b). The trends of the variation in the PL intensities of the porous commercial SiC sample and porous fluorescent SiC samples as the temperature increases from 65 K to 100 K are quite similar. Thus, it is deduced that the passivation induced lower-non-radiative recombination rate can significantly improve the emission efficiency in porous structures. On the other hand, the integrated PL intensity is enhanced in the moderate-temperature region from 100 K to 150 K, which can be partially attributed to the overlap of the enhanced emission peak from the porous layer and the “peak 1” from fluorescent 6H-SiC substrate, which has been discussed in Fig. 3. At the high-temperature region (160–290 K), the activation of non-radiative recombination centers and the escape of carriers from the surface and impurities states lead to thermal quenching of the integrated PL. It should be mentioned here that the emission “peak 1” is manifested at lower temperatures than 150 K, which is related to the DAP recombination via the D-centers in SiC substrates.

**Figure 6:**
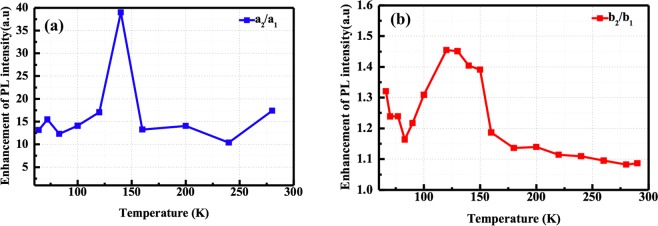
The enhancement of integrated PL intensity in (**a**) porous commercial samples a_2_/a_1_ and (**b**) porous fluorescent samples b_2_/b_1_ as a function of temperature.

To confirm the passivation effect, the temperature-dependent PL measurements were also used to characterize the porous fluorescent SiC without surface passivation. According to Fig. 5(c), the spectral features from the porous surface and the bulk fluorescent SiC are more evident. This is due to less enhancement of emission intensity from the porous structures without surface passivation. Without surface passivation, a huge portion of the photo-generated carriers are captured by non-radiative centers on the porous surface, resulting in fewer remaining carriers to be captured by the radiative centers, yielding weaker emission intensity across the whole temperature range (65–290 K). No obvious abnormal enhancement appears in the integrated PL intensity curve, and the intensity gradually decreases as the temperature increases, as shown in Fig. 5(d). The integrated PL intensity in porous SiC with surface passivation is significantly higher than that without passivation, as shown in Fig. 5(b,d). In addition, the PL intensity of porous layer in fluorescent sample b_2_ is weaker than that of the commercial sample a_2_, due to the thinner thickness of the porous layer in sample b_2_. Figure 6(a,b) show the enhancement of integrated PL intensity in the porous commercial samples and porous fluorescent samples as a function of temperature. The enhancement of integrated PL intensity in the fluorescent samples b_1_/b_2_ is much lower than that of commercial samples a_1_/a_2_, since the integrated value for samples b_1_/b_2_ involves the signal of PL emission from bulk fluorescent SiC. However, it can be deduced that the ALD deposited Al_2_O_3_ can significantly suppress the non-radiative recombination centers and boosts the trapping of carriers at surface radiative states.

**Figure 7:**
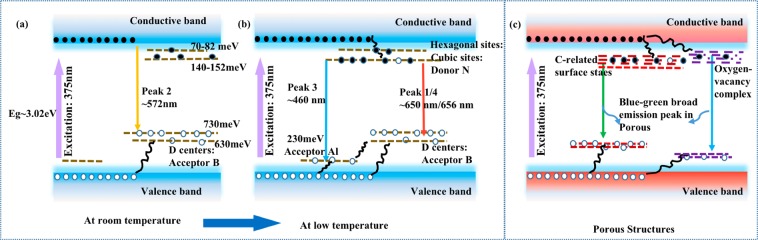
Schematic band diagrams of (**a**) the emission “peak 2” at room temperature; (**b**) the emission “peak 3” and “peak 1/peak 4” at lower temperature. (**c**) The band diagram of surface defects related recombination paths.

A schematic band diagrams are proposed to discuss the observed phenomenon in the temperature dependent PL spectra. The DAP related emission peaks in bulk SiC are depicted in Fig. 7(a,b), while the surface defects-related emission in porous structures is illustrated in Fig. 7(c). Since there are hexagonal-like and cubic-like defect sites in 6H-SiC, the dopants take two kinds of ionization energy levels, which dominate the broad band of N-B DAP recombination^46–48^. However, the N at cubic-like sites with higher binding energy yields more localized excitons and increases the recombination probability without momentum-conserving phonons at low temperature^46^. Therefore, two recombination processes comprise the broad emission of “Peak 1” and “Peak 2”: N donor in cubic-like site and boron-induced double D-centers (D-centers: *E*_*D*1_ = *E*_*v*_ + 0.63 eV and *E*_*D*2_ = *E*_*v*_ + 0.73 eV) and free-to-acceptor recombination^49,50^. At high temperature, the recombination between the phonon-assisted free-electron and boron-induced deep centers (D-center) recombination is the dominant pathway for the emission “Peak 2”^49^. The emission “Peak 1” can be assigned to the emission from N donor to double D-centers^49^, which is optically dominant in DAP emission at low temperature^30,43^. The emission “Peak 3” was observed in both fluorescent and commercial sample, which was explained as being due to Al–N DAP with the unintentionally incorporation of Al atoms induced during the crystal growth^43,44^. The blue-green emission peak in porous structures has been clarified to be origining from non-bridging oxygen hole centers and C-related defect centers located near the interface^23^.

Accordingly, the variation of PL spectra depending on temperature can be referred to the competition between these different recombination paths. Around 100–150 K, an anomalous increase in the integrated PL curve for both passivated samples a_2_ and b_2_ is observed. A similar abnormal increase of PL intensity around 110 K has been reported on Si with Al_2_O_3_ passivation, resulting from the thermal activation of bound excitons into free excitons and the subsequent release of deep-level electrons^51,52^. For porous SiC with ALD passivation, it can be interpreted as the increased carrier mobility and thermal activation of bound excitons, which can enhance the amount of carriers captured by the radiative surface states^30,53^. At higher temperatures (>150 K), the emission via free-to D-centers recombination is dominant and the PL intensity decreases monotonically. Eventually, the emissions from porous structures and fluorescent bulk emission are merged into one broad emission with increasing temperature. At low temperatures, since the concentration of free electrons is much lower than that of electrons bound to N donors, the probability of the DAP recombination (“Peak 1”) is much higher than that via free-electron and the double D-centers (“Peak 2”)^30^. On the other hand, the emission intensity at low temperature from porous structures increases for passivated porous fluorescent SiC. Thus, two prominent emission peaks can be observed in porous fluorescent samples. Since the mobility of the carriers and the activation of the non-recombination centers are closely related to the temperature, the number of carriers captured by the states depends on temperature. For porous structures, the dangling bonds can facilitate the non-radiative recombination through various mid-gap states^31^. From the temperature-dependent measurement results in porous SiC, it can be concluded that the remarkable enhancement of PL intensity in the moderate temperature range is mainly due to the surface passivation effect of ALD Al_2_O_3_ films.

### Low temperature Time-resolved PL spectra

**Figure 8:**
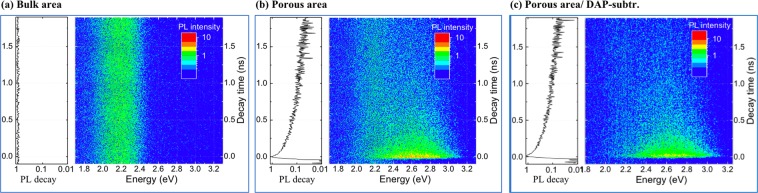
Time-resolved PL mapping of the bulk fluorescent SiC (**a**) and porous sample b_2_ (**b**); (**c**) The map obtained from the PL map shown in Fig. 6(b) by subtracting the “time-constant” DAP-signal. The frequency-integrated time-resolved PL transient spectra (left: with logarithmic scaling) are shown in each figure.

To analyze the decay features, time-resolved PL maps were measured to characterize the porous fluorescent SiC. The samples were excited by 100 fs pulses (375 nm) produced by frequency-doubled output of a Ti:sapphire laser combined with a 1.9 MHz pulse picker (Coherent Mira). The excitation spot size through an inverted microscope (Zeiss Axiovert 100) was around 50 µm due to a long (5 cm) focal distance lens. Time-resolved PL mapping data at room temperature were collected by a streak-camera (C5680, Hamamatsu) equipped with a polychromator. The instrument response function is around 10 ps. The results of porous and bulk areas of the sample b_2_ are shown in Fig. 8. At room temperature, the bulk fluorescent SiC exhibits a long lifetime of free-to D-centers emission centered at 572 nm (~2.19 eV). The time-resolved PL mapping in the porous area reveals a weak long-lifetime component, which is similar to the bulk area PL, and a short-lifetime PL component at the broad-blue flank. The decay curves obtained by integrating over the whole spectral region are also displayed in Fig. 8. The time-resolved fluorescent data of the porous sample b_2_ with subtracted free to D-centers emission signal reveals that the decay curve is mainly attributed to the short-lifetime component from porous structures, as shown in Fig. 8(c). Since the injection of excitation is extremely low and the carrier lifetime for bulk fluorescent SiC is rather long (in the range of ms)^22^, the time-resolved PL decay curves mainly reveal the signal from porous structures. One important feature becomes clear by comparing the decay mappings: the overall carrier decay of porous fluorescent SiC is quite fast, which is mainly attributed by the high density of internal/surface defects.

The mapping results clearly showed the relative decay rates for different emission mechanisms in porous structures and fluorescent bulk. In this case, a further discussion can to be carried for the temperature dependent of the fast decay component associated with the surface emission in porous structures. The time-resolved PL spectra of porous samples b_1_ and b_2_ were measured at 65 K and 290 K, utilizing a pulsed diode laser (375 nm, pulse width ~44 ps, 0.74 mW) as the excitation source coupled into a microscope. The picosecond pulsed diode laser was controlled by a programmable laser driver (LDH-D-C-375 with PDL 820, PicoQuant GmbH). The prescaler/divider and sequencer/splitter in the laser driver can control the output frequency from the base frequency (50 MHz, 64 MHz, and 80 MHz) provided by the base oscillator. The emission light was filtered by an optical cutoff filter (long pass filter 405 nm). The signal was acquired by a photon detector-photomultiplier tube (PMT) and time correlated single photon counting (TCSPC) system. The fastest decay component here is also probably affected by the instrument response function (FWHM~ 210 ps).

**Figure 9:**
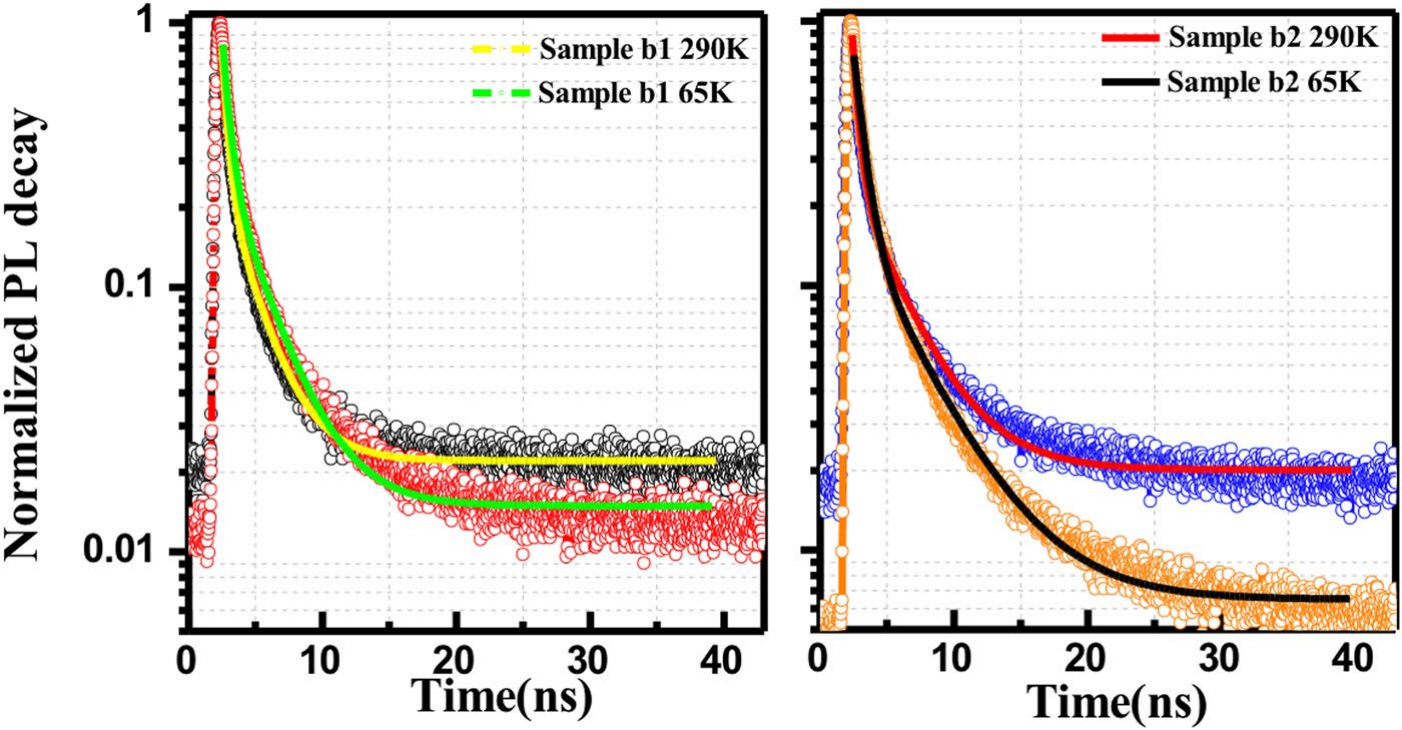
Normalized time-resolved PL of porous fluorescent SiC before (sample b_1_) and after surface passivation (sample b_2_), measured at 290 K and 65 K. The decay curves were fitted with a double-exponential decay function.

The decay acquired here is a cumulative effect of emission peaks from porous structures and deep structure defects or non-radiative surface states. The luminescence decay in Fig. 9 can be adequately described by double-exponential fits. The mechanisms of lifetime quenching are revealed by the lifetimes of the respective double-exponential fitting, including the radiative surface recombination from different surface defect centers in the porous structures. The amplitude-weighted lifetimes were calculated by using the following relation:

$${\boldsymbol{I}}({\boldsymbol{t}})=\mathop{\sum }\limits_{{\boldsymbol{i}}=1}^{{\boldsymbol{n}}}{{\boldsymbol{A}}}_{{\boldsymbol{i}}}{{\boldsymbol{e}}}^{-\frac{\tau }{{\tau }_{{\boldsymbol{i}}}}}$$

The calculated lifetime components ***τ***_*i*_ and weights of each component *A*_*i*_ (i = 1, 2) are listed in Table 1. The explanation for the carrier dynamics of ***τ***_*1*_, and ***τ***_*2*_ are related to structure defects in fluorescent SiC near surface (mixed with non-radiative recombination centers^54^), neutral oxygen vacancies and interface C-related surface defects that are generated during anodic oxidation process^34,38,39^.

**Table 1:** Lifetimes in the time-resolved PL decay fitted by double exponential components.

Samples	τ_2_ (ns)	A_2_	τ_1_(ns)	A_1_
Sample b_1_ (290 K)	2.26	0.69	0.39	209.56
Sample b_1_ (65 K)	2.76	0.67	0.48	101.45
Sample b_2_ (290 K)	3.37	0.46	0.44	147.61
Sample b_2_ (65 K)	4.21	0.28	0.74	17.54

The carrier lifetime for the fastest recombination channel related to deep structure defects is 0.39 ns and 0.48 ns for porous SiC without passivation at 290 K and 65 K, respectively, *i.e*. the non-radiative component was suppressed at lower temperature. A similar tendency has been found in the passivated porous fluorescent and porous commercial SiC samples. The carrier lifetime **τ**_2_ associated with the neutral oxygen vacancies and interface C-related surface defects increases after surface passivation, especially at low temperature. The ALD passivated porous sample has a slower PL decay, providing direct evidence for the significant reduction of surface non-radiative centers. This result strongly supports the prominent enhancement of PL intensity in porous fluorescent SiC, especially at low temperature.

## Conclusions

In summary, we have revealed the temperature-dependent PL properties of porous fluorescent SiC. An impressive enhancement of PL intensity was observed in porous SiC with ALD Al_2_O_3_ film, especially at low temperatures. There are two prominent emission peaks from porous fluorescent SiC at low temperatures. The broad emission peak at 650 nm from bulk layer can be attributed to the DAP recombination via N donor to boron related double D-centers, at a temperature lower than 150 K. The other broad emission peak located at 517 nm has been attributed to non-bridging oxygen hole centers and C-related defect centers located near the interface in porous structures. At high temperatures above 150 K, the recombination between the phonon-assisted free-electron and boron-induced D-centers is the dominant pathway for the emission. At lower temperatures below 150 K, passivation caused an abnormal increase of PL intensity with increasing temperature. The enhanced carrier lifetime as visualized by the time-resolved PL and significantly increased PL intensity provide strong experimental evidence of the passivation effect on the surface non-radiative recombination centers. This work represents a step forward in understanding of the optical properties of porous fluorescent SiC for white LED applications.

## Data Availability

The datasets generated during and/or analyzed during the current study are available from the corresponding author on reasonable request.

## References

[1] Eddy C, Gaskill D (2009). Silicon carbide as a platform for power electronics. Science.

[2] Morkoc H (1994). Large-band-gap SiC, III-V nitride, and II-VI ZnSe-based semiconductor device technologies. J. Appl. Phys..

[3] Palmour JW, Edmond JA, Kong HS, Carter CH (1993). 6H-silicon carbide devices and applications. Physica B: Condensed Matter.

[4] Santavirta S (1998). Biocompatibility of silicon carbide in colony formation test *in vitro*. Arch. Orthop. Traum. Su..

[5] Oliveros A, Guiseppi-Elie A, Saddow SE (2013). Silicon carbide: a versatile material for biosensor applications. Biomed. Microdevices.

[6] Li X (2005). Micro/nanoscale mechanical and tribological characterization of SiC for orthopedic applications. J. Biomed. Mater. Res. B.

[7] Rosenbloom AJ (2004). Nanoporous SiC: A candidate semi-permeable material for biomedical applications. Biomed. Microdevices.

[8] Beke D (2013). Silicon carbide quantum dots for bioimaging. J. Mater. Res..

[9] Godignon, P. SiC materials and technologies for sensors development. *Mater. Sci. Forum*, Trans Tech Publ, pp. 1009–1014 (2005).

[10] Naderi N, Hashim M (2013). Visible-blind ultraviolet photodetectors on porous silicon carbide substrates. Mater. Res. Bull..

[11] Dezauzier C (1995). Electrical characterization of SiC for high-temperature thermal-sensor applications. Sens. Actuators A: Phys..

[12] George, M., Ayoub, M., Ila, D. & Larkin, D. Elevated temperature silicon carbide chemical sensors, *MRS Proceedings, Cambridge Univ Press*, pp. 123 (1999).

[13] Saddow S, Mynbaeva M, Smith M, Smirnov A, Dimitriev V (2001). Growth of SiC epitaxial layers on porous surfaces of varying porosity. Appl. Surf. Sci..

[14] Inoki C (2003). Growth of GaN on porous SiC and GaN substrates. Phys. Status Solidi A.

[15] Yun F (2002). Growth of GaN films on porous SiC substrate by molecular-beam epitaxy. Appl. Phys. Lett..

[16] Matsumoto T (1994). Blue-green luminescence from porous silicon carbide. Appl. Phys. Lett..

[17] Nishimura T (2010). High efficiency violet to blue light emission in porous SiC produced by anodic method. Phys. Status Solidi C.

[18] Petrova-Koch V (1995). Luminescence enhancement by electrochemical etching of SiC (6H). Thin Solid Films.

[19] Jessensky O, Müller F, Gösele U (1997). Microstructure and photoluminescence of electrochemically etched porous SiC. Thin Solid Films.

[20] Lee K-H, Lee S-K, Jeon K-S (2009). Photoluminescent properties of silicon carbide and porous silicon carbide after annealing. Appl. Surf. Sci..

[21] Rittenhouse TL (2004). Surface-state origin for the blueshifted emission in anodically etched porous silicon carbide. J. Appl. Phys..

[22] Kamiyama S (2011). Fluorescent SiC and its application to white light-emitting diodes. J. Semicond..

[23] Lu W (2017). White Light Emission from Fluorescent SiC with Porous Surface. Sci. Rep..

[24] Lu W (2017). Effective optimization of surface passivation on porous silicon carbide using atomic layer deposited Al_2_O_3_. RSC Adv..

[25] Fan J, Li H, Wang J, Xiao M (2012). Fabrication and photoluminescence of SiC quantum dots stemming from 3C, 6H, and 4H polytypes of bulk SiC. Appl. Phys. Lett..

[26] Beke D (2017). Harnessing no-photon exciton generation chemistry to engineer semiconductor nanostructures. Sci Rep.

[27] Alekseev SA, Zaitsev VN, Botsoa J, Barbier D (2007). Fourier transform infrared spectroscopy and temperature-programmed desorption mass spectrometry study of surface chemistry of porous 6H-SiC. Chem. Mater..

[28] Rashid M, Horrocks BR, Healy N, Goss JP, Horsfall AB (2016). Optical properties of mesoporous 4H-SiC prepared by anodic electrochemical etching. J. Appl. Phys..

[29] Lu T (2014). Temperature-dependent photoluminescence in light-emitting diodes. Sci. Rep..

[30] Reshchikov MA (2014). Temperature dependence of defect-related photoluminescence in III-V and II-VI semiconductors. J. Appl. Phys..

[31] Gfroerer, T. H. Photoluminescence in Analysis of Surfaces and Interfaces. *Encyclopedia of analytical chemistry: applications, theory and instrumentation* (2006).

[32] Botsoa J (2007). Photoluminescence of 6H–SiC nanostructures fabricated by electrochemical etching. J. Appl. Phys..

[33] Shishkin Y, Choyke WJ, Devaty RP (2004). Photoelectrochemical etching of n-type 4H silicon carbide. J. Appl. Phys..

[34] Konstantinov AO, Harris CI, Janzén E (1994). Electrical properties and formation mechanism of porous silicon carbide. Appl. Phys. Lett..

[35] Shimoda K, Park J-S, Hinoki T, Kohyama A (2007). Influence of surface structure of SiC nano-sized powder analyzed by X-ray photoelectron spectroscopy on basic powder characteristics. Appl. Surf. Sci..

[36] Jensen H, Soloviev A, Li Z, Søgaard EG (2005). XPS and FTIR investigation of the surface properties of different prepared titania nano-powders. Appl. Surf. Sci..

[37] Gat E (1992). A study of the effect of composition on the microstructural evolution of a–Si x C 1− x: H PECVD films: IR absorption and XPS characterizations. J. Mater. Res..

[38] Nikas V (2014). The origin of white luminescence from silicon oxycarbide thin films. Appl. Phys. Lett..

[39] Tabassum N (2016). Time-resolved analysis of the white photoluminescence from chemically synthesized SiCxOy thin films and nanowires. Appl. Phys. Lett..

[40] Gallis S, Nikas V, Suhag H, Huang M, Kaloyeros AE (2010). White light emission from amorphous silicon oxycarbide (a-SiC x O y) thin films: Role of composition and postdeposition annealing. Appl. Phys. Lett..

[41] Ghobadi TU, Ghobadi A, Okyay T, Topalli K, Okyay A (2016). Controlling luminescent silicon nanoparticle emission produced by nanosecond pulsed laser ablation: role of interface defect states and crystallinity phase. RSC Adv..

[42] Konstantinov AO, Henry A, Harris CI, Janzén E (1995). Photoluminescence studies of porous silicon carbide. Appl. Phys. Lett..

[43] Kamiyama S (2006). Extremely high quantum efficiency of donor-acceptor-pair emission in N-and-B-doped 6H-SiC. J. Appl. Phys..

[44] Edmond J, Kong H, Suvorov A, Waltz D, Carter C (1997). 6H‐Silicon Carbide Light Emitting Diodes and UV Photodiodes. Phys. Status Solidi A.

[45] Choyke W.J. (1969). OPTICAL PROPERTIES OF POLYTYPES OF SiC: INTERBAND ABSORPTION, AND LUMINESCENCE OF NITROGEN-EXCITON COMPLEXES. Silicon Carbide–1968.

[46] Kimoto T (1995). Nitrogen donors and deep levels in high-quality 4H–SiC epilayers grown by chemical vapor deposition. Appl. Phys. Lett..

[47] Ikeda M, Matsunami H, Tanaka T (1980). Site effect on the impurity levels in 4H, 6H and 15R SiC. Phys. Rev. B.

[48] Raynaud C, Ducroquet F, Guillot G, Porter LM, Davis RF (1994). Determination of ionization energies of the nitrogen donors in 6H‐SiC by admittance spectroscopy. J. Appl. Phys..

[49] Gavryushin V (2014). Examination of Photoluminescence Temperature Dependencies in N-B Co-doped 6H-SiC. IOP Conf. Ser.: Mater. Sci. Eng..

[50] Suttrop W, Pensl G, Lanig P (1990). Boron-related deep centers in 6H-SiC. Appl. Phys. A.

[51] Sun E, Su F-H, Chen C-H, Chen M-J (2010). Enhancement of photoluminescence intensity from Si nanodots using Al_2_O_3_ surface passivation layer grown by atomic layer deposition. Appl. Surf. Sci..

[52] Chen M, Shih Y, Wu M, Tsai F (2007). Enhancement in the efficiency of light emission from silicon by a thin Al_2_O_3_ surface-passivating layer grown by atomic layer deposition at low temperature. J. Appl. Phys..

[53] Arora ND, Hauser JR, Roulston DJ (1982). Electron and hole mobilities in silicon as a function of concentration and temperature. IEEE Trans Electron Devices.

[54] Cantin J-L (2004). Identification of the Carbon Dangling Bond Center at the 4H−SiC/SiO_2_ Interface by an EPR Study in Oxidized Porous SiC. Phys. Rev. Lett..

